# Natural Potent NAAA Inhibitor Atractylodin Counteracts LPS-Induced Microglial Activation

**DOI:** 10.3389/fphar.2020.577319

**Published:** 2020-10-02

**Authors:** Longhe Yang, Chunyan Ji, Yitian Li, Fan Hu, Fang Zhang, Haiping Zhang, Long Li, Jie Ren, Zhaokai Wang, Yan Qiu

**Affiliations:** ^1^ Technical Innovation Center for Utilization of Marine Biological Resources, Third Institute of Oceanography, Ministry of Natural Resources, Xiamen, China; ^2^ Eye Institute of Xiamen University, Fujian Provincial Key Laboratory of Ophthalmology and Visual Science, School of Medicine, Xiamen University, Xiamen, China; ^3^ Center for High Performance Computing, Joint Engineering Research Center for Health Big Data Intelligent Analysis Technology, Shenzhen Institutes of Advanced Technology, Chinese Academy of Sciences, Shenzhen, China; ^4^ Institute of Drug Discovery Technology, Ningbo University, Ningbo, China

**Keywords:** NAAA inhibitor, atractylodin****, anti-inflammation, microglia, traditional Chinese medicine

## Abstract

*N*-acylethanolamine-hydrolyzing acid amidase (NAAA) is a lysosomal enzyme that inhibits the degradation of palmitoylethanolamide (PEA), an endogenous lipid that induces analgesic, anti-inflammation, and anti-multiple sclerosis through PPARα activation. Only a few potent NAAA inhibitors have been reported to date, which is mainly due to the restricted substrate-binding site of NAAA. Here, we established a high-throughput fluorescence-based assay for NAAA inhibitor screening. Several new classes of NAAA inhibitors were discovered from a small library of natural products. One of these is atractylodin, a polyethylene alkyne compound from the root of *Atractylodes lancea* (Thunb) DC., which significantly inhibits NAAA activity and has an IC_50_ of 2.81 µM. Kinetic analyses and dialysis assays suggested that atractylodin engages in competitive inhibition *via* reversible reaction to the enzyme. Docking assays revealed that atractylodin occupies the catalytic cavity of NAAA, where the atractylodin furan head group has a hydrophobic-related interaction with the backbone of the Trp181 and Leu152 residues of human NAAA. Further investigation indicated that atractylodin significantly increases PEA and OEA levels and dose-dependently inhibits LPS-induced nitrate, TNF-α, IL-1β, and IL-6 pro-inflammatory cytokine release in BV-2 microglia. Our results show that atractylodin elevates cellular PEA levels and inhibits microglial activation by inhibiting NAAA activity, which in turn could contribute to NAAA functional research.

## Introduction

*N*-Acylethanolamines (NAEs) are a class of functional long-chain lipids that are ubiquitous in animal tissues including arachidonyl ethanolamide (anandamide, AEA), oleoylethanolamide (OEA), and *N*-palmitoylethanolamide (PEA) (Iannotti et al., 2016). NAEs are widely involved in modulating various physiological responses ranging from inflammation, pain, neuroprotection, appetite, and lipid metabolism (Kaur et al., 2016). PEA is currently considered an anti-inflammatory and analgesic molecule that interacts with peroxisome proliferator-activated receptor-α (PPAR-α) (Pontis et al., 2016). However, PEA concentrations decreased during various pathological conditions such as in that spinal cord after chronic constriction injury (CCI) (Petrosino et al., 2007) or in hind paw tissues after application of complete Freund’s adjuvant (Bonezzi et al., 2016). Thus, pharmacological tools that can selectively modulate PEA levels have become a recent research topic of interest.

*N*-acylethanolamine-hydrolyzing acid amidase (NAAA) is the major enzyme responsible for the inactivation of PEA to ethanolamine and the corresponding palmitic acid (Tsuboi et al., 2005; Tsuboi et al., 2007a; Ueda et al., 2010). NAAA was first cloned by Sun et al. in 2005 and belongs to choloylglycine hydrolase family (Tsuboi et al., 2005). NAAA is mainly distributed in lysosomes and shows no homology to fatty acid amide hydrolase (FAAH), another important NAE degradation enzyme (Tsuboi et al., 2007b; Ren et al., 2020). Several series of compounds targeting NAAA inhibition have been reported to have potential therapeutic use for inflammatory and neuropathic pain (Yang et al., 2015; Petrosino et al., 2017; Ren et al., 2017), multiple sclerosis (Migliore et al., 2016), allergic dermatitis (Sasso et al., 2018), arthritis (Bonezzi et al., 2016), and inflammatory bowel diseases (Alhouayek et al., 2015).

However, the number of reported NAAA inhibitors remains limited and is mainly due to the restricted substrate-binding site of NAAA. The catalytic cavity of NAAA is suitable for accommodating only narrow and relatively straight compound backbones (Gorelik et al., 2018). Furthermore, identifying specific molecular structures to fit the catalytic center of this enzyme remains a challenge for synthetic chemists.

Plant-based natural molecules are promising and emerging therapeutic alternatives to inflammation and pain. Petrosino and colleagues (2015) reported a compound named diacerin, which was modified from the natural product rhein and had an IC_50_ value of 7.2 μM on NAAA inhibition. This compound also inhibits inflammation by elevating endogenous PEA levels in an acute inflammatory pain rat model that received an intraplantar injection of carrageenan (Petrosino et al., 2015). We also reported a marine carotenoid named fucoxanthinol from brown seaweeds that inhibited NAAA activity with an IC_50_ value of 12.75 μM and significantly attenuated the expression of inflammatory factors through the NAAA-PEA pathway in LPS-induced RAW264.7 cells (Jin et al., 2020). Therefore, screening and discovery of adapting chemical groups from natural products would provide a rich resource for lead compounds for the development of novel NAAA inhibitors.

The aim of the present study was to identify novel small molecules from natural products that selectively and effectively inhibit NAAA. NAAA activity was measured by hydrolyzing a fluorogenic compound N-(4-methyl coumarin) palmitamide (PAMCA) to 4-methyl coumarin (West et al., 2012a; West et al., 2012b). We screened a small natural compound library consisting of 465 diverse natural compounds from traditional Chinese medicine (TCM), food resources, and marine derivatives collected by our laboratory. Atractylodin from the root of *Atractylodes lancea* (Thunb) DC. (*A. Rhizoma*) was identified as the most potent NAAA inhibitor in this study. Sesamin, d-tetrandrine, amentoflavone, and anwuligan also indicated weak NAAA inhibitory activity. Moreover, atractylodin suppressed inflammatory cytokines in lipopolysaccharides (LPS)-induced BV2 microglial cells in a dose-dependent manner by increasing endogenous PEA and OEA.

## Materials and Methods

### Chemicals and Reagents

Natural compounds (≥ 98% purity) were purchased from Purechem-Standard Co., Ltd. (Chengdu, China). Lipopolysaccharides (LPS) from *Escherichia coli* O111:B4 were purchased from Sigma-Aldrich (Shanghai, China). Cell counting kit-8 (CCK-8) was purchased from Dojindo (Shanghai, China). Griess reagent kit for nitrite determination was purchased from ThermoFisher (Shanghai, China). TNF-α, IL-1β, and IL-6 Valukine ELISA kits and recombinant human NAAA protein were purchased from R&D Systems (Shanghai, China).

### NAAA Inhibitors Screening

Fluorometric NAAA activity measurement was used to evaluate the natural products inhibitory potency towards NAAA as previously reported with minor modifications (West et al., 2012a; West et al., 2012b). Briefly, in our initial screening, recombinant human NAAA protein was diluted in NAAA buffer (3 mM DTT, 0.1% Triton X-100, 0.05% BSA, 150 mM NaCl, pH 4.5) to 0.25 µg/ml and then 20 µL were transferred to a 96-well half-volume black plate followed by the addition of 2 µL of DMSO-diluted compounds at a final concentration of 50 µM. After shaking for 10 min on a shaking plate, 28 µL of the N-(4-methylcoumarin)-palmitamide (PAMCA) substrate in assay buffer (final concentration: 25 µM) were added, and the reaction was allowed to proceed at 37˚C for 30 min, then enzyme activity was monitored at a wavelength of 460 nm (excitation wavelength: 360 nm) on a multimode reader (Mithras LB 943, Berthold, Germany). Compounds showing > 50% inhibition were subjected to a series of two-fold dilutions for IC_50_ testing.

### Molecular Modeling

Docking of the atractylodin to the NAAA active site was performed using Autodock Vina{Trott, 2010 #276} and the results were visualized by Discovery Studio Visualizer. The three-dimensional model of NAAA based on its crystal structure (PDB ID: 6DXX; Gorelik et al., 2018) was used in molecular modeling. The scripts named “prepare_receptor4.py” and “prepare_ligand4.py” from AutoDockTools were used for preparing AutoDock vina input files, respectively. The exhaustiveness was set to 8, the num_modes was set to 20, and energy_range was set to 3. The active binding site region was based on known ligand (ARN19702) position from the crystal structure. The docking region was set to include the active binding site, with size around 35, 35, 35Å and center around coordinate of (30.70 Å, 1.66 Å, 26.24 Å) in x, y, z.

### Molecular Dynamic Stimulation Analysis

The AMBER-99SB force field was used to run molecular dynamic (MD) simulation for the NAAA-Atractylodin complex by Gromacs program. The ACPYPE tool, which relies on Antechamber, was used to generate the topology of ligand and the partial charges of ligand. A dodecahedron box was used, and the target-ligand complex was placed at the center. The size of dodecahedron box is defined based on a minimum distance of 1 nm from the protein to box edge. The TIP3P water molecules was used to filled the dodecahedron box, the counter ions was added to neutralize the total charge of the system using the Gromacs program tool. The long-range electrostatic interactions under the periodic boundary conditions was calculated with Particle Mesh Ewald approach. A cutoff of 14 Å was used for van der Waals non-bonded interactions. LINCS algorithm was applied to constrain the covalent bonds involving hydrogen atoms. MD simulation was performed and energy minimization step-size was 0.001ns. Followed with a 100 ps simulation with isothermal-isovolumetric ensemble (NVT) for water equilibrium, and 10ns simulation with isothermal-isobaric ensemble (NPT) for system equilibrium. After that, a 100ns NPT production simulation (step size 2 fs) was carried out. During the simulation, a fixed temperature of 308 K and a pressure of 1 atm were maintained by Parrinello-Rahmanbarostat and the modified Berendsen thermostat. The RMSD and hydrogen bond number of the trajectory was calculated using Gromacs tools.

### BV2 Cell Culture and Treatment

The microglia cell line BV-2 was cultured in DMEM medium containing 10% fetal bovine serum and antibiotics (100 U/ml penicillin, 100 g/ml streptomycin) and maintained in a humidified 5% CO_2_ incubator at 37°C. For the experiment, cells were seeded overnight into a 24-well plate for the cytokine assay or into 100-mm dishes for NAE extraction and quantification. The next day, the cells were incubated with fresh culture medium containing the indicated concentration of atractylodin for 0.5 h and following lipopolysaccharide (LPS) treatment (1 μg/ml). Cells incubated with vehicle only (DMSO, 0.1%) were used as control.

### FAE Extraction and Quantification

FAEs were extracted from BV-2 cells as previously reported (Yang et al., 2015). Briefly, cells were harvested and ultrasonicated in 2 ml of methanol/water (1:1, vol/vol) containing 100 pmoL of heptadecenoylethanolamide as internal standard. Lipids were extracted using 4 ml of chloroform, and the organic phases were collected, and dried under N_2_. Lipids were dissolved in 1 ml chloroform and eluted in a silica column. The extract containing FAEs was eluted with methanol/chloroform (v/v, 1/9) and dried under N_2_, and then redissolved in 100 μl of methanol for HPLC-MS/MS analysis. An ABI 3200 Q-Trap mass spectrometer (Applied Biosystems, Concord, Canada equipped with a 1100-LC system (Agilent, Shanghai, China) was used in this experiment. The precursor/product ion transitions in multiple reaction monitoring mode (MRM) were used for mass analysis and quantitation. The molecular ions were monitored at *m/z* 300.2/62.0 for PEA, *m/z* 326.1/62.0 for OEA, *m/z* 348.00/62.00 for AEA, *m/z* 379.10/287.10 for 2-AG, and *m/z* 313.1/62.0 for C17:1 FAE.

### Nitrite Quantification

The concentration of nitrite in the culture medium was determined using a Griess reagent kit that reflected the amount of NO released by cultured cells. Culture supernatants (75 μl) were reacted with an equal volume of Griess reagent kit for 30 min at room temperature, and the absorbance of the diazonium compound at a wavelength of 560 nm was measured.

### Data Analysis

Statistical analysis was performed using PRISM 5 software. The results were expressed as mean ± standard error of the mean (SEM). The Student’s *t* test and one-way ANOVA were used for comparisons between and among different groups.

## Results

### Atractylodin Is a Potent NAAA Inhibitor

**Figure 1:**
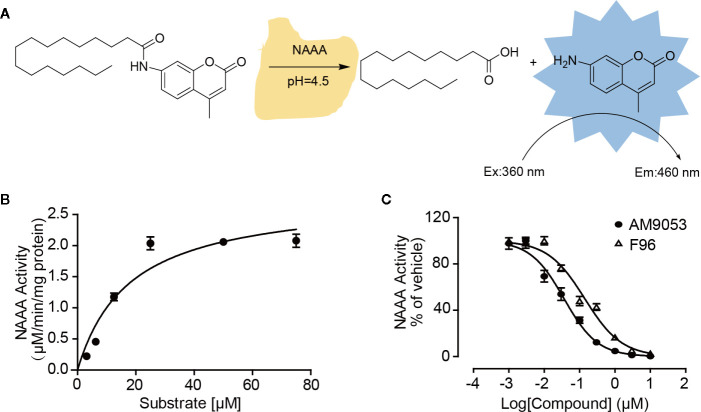
Screening model of NAAA inhibitors based on fluorescence. **(A)** Illustrator of the hydrolysis of the PAMCA substrate into the fluorescent compound AMC by NAAA. **(B)** Kinetic assay of NAAA with PAMCA. **(C)** Concentration-dependent inhibition of NAAA by AM9053 and F96. The data are expressed as mean ± SEM.

Although several compounds have been discovered as NAAA inhibitors, reports on natural derivatives are limited. Here, we constructed a small library of 465 natural compounds consisting of alkaloids, flavonoids, coumarins, terpenoids, polyacetylenes, and aliphatics for activity screening. First, we established a high-throughput screening system for NAAA inhibitors using the fluorogenic compound N-(4-methyl coumarin) palmitamide (PAMCA) as hydrolyzed substrate (West et al., 2012b). PAMCA was metabolized to the fluorescent 7-amino-4-methyl coumarin (AMC) and palmitic acid by NAAA at pH 4.5 (**Figure 1A**). In this assay, the enzyme hydrolyzed PAMCA with an apparent K_m_ value of 17.92 ± 3.54 µM (**Figure 1B**). To further confirm the effectiveness of the screening system, another two reported potent NAAA inhibitors, F96 and AM9053, were tested. The results showed that these compounds inhibited NAAA activity with IC_50_ values of 140.3 nM and 36.4 nM, respectively (**Figure 1C**). These results closely match the reported data with the IC_50_ values of 270 nM for F96 (Yang et al., 2015), and 30 nM for AM 9053 (West et al., 2012a).

**Figure 2:**
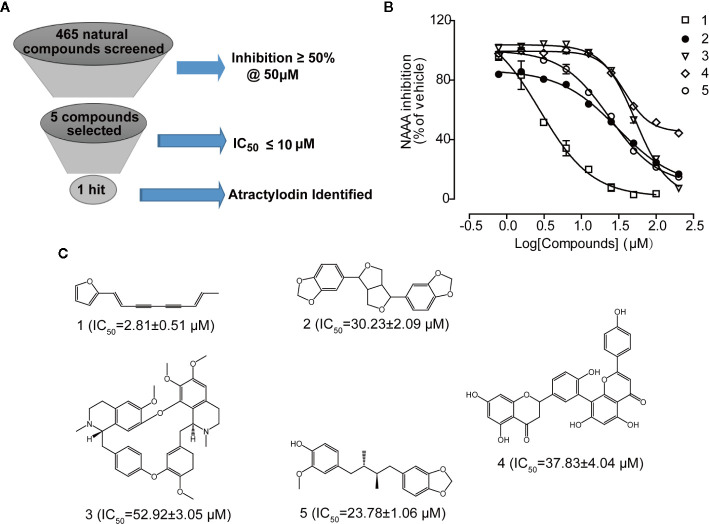
Atractylodin screened from a small natural compound library as potent NAAA inhibitor. **(A)** Five compounds screened from 465 compounds showing NAAA inhibition > 50% at 50 μM. **(B)** Concentration-dependent inhibition of NAAA activity by compounds 1–5, and **(C)**. Chemical structure of natural NAAA inhibitors. 1. Atractylodin, 2. Sesamin, 3. D-Tetrandrine, 4. Amentoflavone, 5. Anwuligan.

NAAA screening was conducted on the natural products library with 465 compounds at 50 µM, and then compounds with more than 50% inhibition were selected. As shown in **Figure 2**, five natural compounds were selected in our primary screening, which inhibited NAAA activity in a dose-dependent manner and with an IC_50_ between 2.81 µM and 52.92 µM (**Figure 2B**). Polyethylene alkyne compound atractylodin (1) (**Figure 2C**), isolated from *A. rhizoma*, was identified as the most potent NAAA inhibitor in this screening. The other four natural products sesamin (2), D-tetrandrine (3), amentoflavone (4), and anwuligan (5) exhibited relatively weak activity against NAAA. Atractylodin was selected to explore the binding characteristics to NAAA.

### Atractylodin Is a Rapid, Reversible, and Competitive NAAA Inhibitor

**Figure 3:**
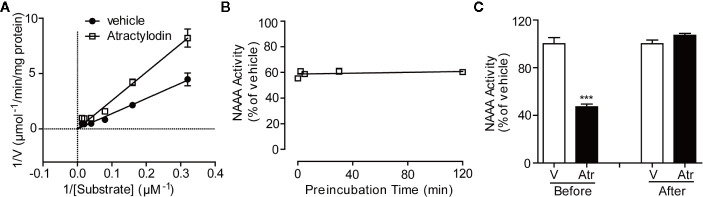
Characterization of the NAAA inhibitor atractylodin. **(A)** Lineweaver-Burk plot indicates competitive inhibition of NAAA by atractylodin (Atr, 3 µM). **(B)** Rapid, **(C)** Dilution assay indicates Atr, a reversible NAAA inhibitor. Data are shown as the mean ± SEM. ***P < 0.001 compared with the control group (V).

To characterize the detailed mechanism of atractylodin with NAAA, we first conceived kinetic analyses and dialysis assay. The Lineweaver-Burk plot of kinetic analyses revealed that atractylodin blocked NAAA through a competitive mechanism (**Figure 3A**). Time-course experiments showed that the inhibition activity was rapid and unaffected by longer pre-incubation of hNAAA with atractylodin (**Figure 3B**). Moreover, in the dialysis experiment, NAAA activity was fully recovered after dialysis with the NAAA-atractylodin mixture in PBS buffer (**Figure 3C**), which suggests reversible inhibition. Taken together, these results indicate that atractylodin is a rapid, reversible, and competitive NAAA inhibitor.

### Atractylodin Occupies the Catalytic Cavity of NAAA

**Figure 4:**
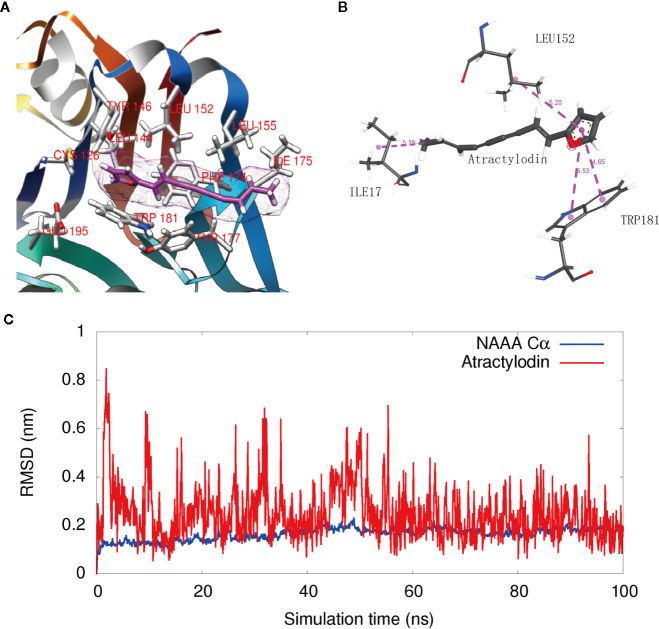
Interaction of NAAA with atractylodin. **(A)** Docking detail of the substrate-binding site of human NAAA (PDB ID: 6DXX) bound to the inhibitor atractylodin (light pink carbons). **(B)** The 2D Schematic diagram of the interaction. **(C)** The RMSD fluctuation of NAAA Cα atoms, and the RMSD fluctuation of Atractylodin along the 100 ns MD simulations.

To visualize the enzyme active site modified by atractylodin, we constructed a docking model based on the protein structure reported by Gorelik et al. (2018). Docking studies showed that atractylodin occupied the catalytic cavity of NAAA, where the furan head group of atractylodin has a hydrophobic-related interaction with the backbone of the TRP181 and LEU152 residues of human NAAA (**Figures 4A, B**). In detail, two Pi-Pi stack interactions have been formed between the furan group in Atractylodin and ring of benzpyrole in TRP181, with distance of 4.65Å and 5.53 Å respectively. The furan group also formed a Pi-Alkyl interaction with LEU152 with a distance of 5.2 Å. Simultaneously, there is a methyl interaction between furan with ILE 175 with a distance of 5.15 Å at the entrance of the pocket, which enabled the accommodation of the natural product in the cavity without falling out easily. Atractylodin occupied the substrate-binding cavity, preventing the entrance of PEA, resulting in NAAA inhibition.

**Figure 4C** shows that the ligand fluctuation is relatively small with RMSD value round 0.2 nm in the last 40 ns (red). The result indicates that the binding is very stable and the simulation achieves relative convergences. The RMSD fluctuation of NAAA Cα atoms are also small (below 0.2 nm for most of the time), indicating no large conformation change of the NAAA protein during the simulation.

### NAAA Inhibition Reduces Nitrite Oroduction in LPS-induced BV-2 Microglial Cells

**Figure 5:**
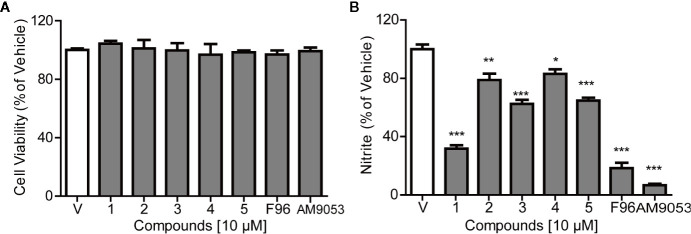
Effects of NAAA inhibitors on cell viability and nitrite expression in BV-2 cells. **(A)** Cell viability and **(B)** The inhibitory effect of compounds on nitrite production. Numbers represent Sesamin (1), Atractylodin (2), D-tetrandrine (3), amentoflavone (4), and anwuligan (5). Data are shown as mean ± SEM. *P < 0.05, **P < 0.01, ***P < 0.001 compared with the control group (V).

The cytotoxicity of the NAAA inhibitors on BV-2 cells was assessed using CCK-8. **Figure 5A** shows that the selected natural products and synthetic potent NAAA inhibitors F96 and AM9053 did not exhibit any detectable cytotoxic effects. NAAA is highly expressed in immune cells, including tissue macrophages, monocytes, and microglia. Previous studies have shown that pharmacological inhibition of NAAA reduces the expression of inflammatory cytokines IL-1β, TNF-α, IL-6, and inflammatory mediates nitrite in RAW264.7 cells (Alhouayek et al., 2017). In this context, we also tested the effects of selected natural compounds together with synthetic compounds on LPS-induced nitrate secretion in BV-2 cells. The inhibitory activity of the natural products against nitrite release were first investigated in LPS-induced BV-2 cells. Microglia were pre-incubated with the compounds at a concentration of 10 µM for 30 min, and LPS was added for another 24 h. The concentrations of nitrite were monitored using the Griess reagent kit. In this setting, all compounds decreased LPS-induced nitrate production (**Figure 5B**). Among these compounds, the potent NAAA inhibitors F96 and AM9053 respectively decreased nitrate production by 81.54 ± 7.34% and 93.2 ± 1.45% compared with vehicle group (**Figure 5B**). Atractylodin (1) significantly reduced nitrite production by 68.21%, and the other natural chemicals sesamin (2), D-Tetrandrine (3), amentoflavone (4), and anwuligan (5) suppressed the LPS-induced nitrite production between 22.15% and 37.44%, which was associated with NAAA inhibitory activity.

### Atractylodin Inhibits Inflammatory Factors Generated in LPS-induced BV-2 Cells

**Figure 6:**
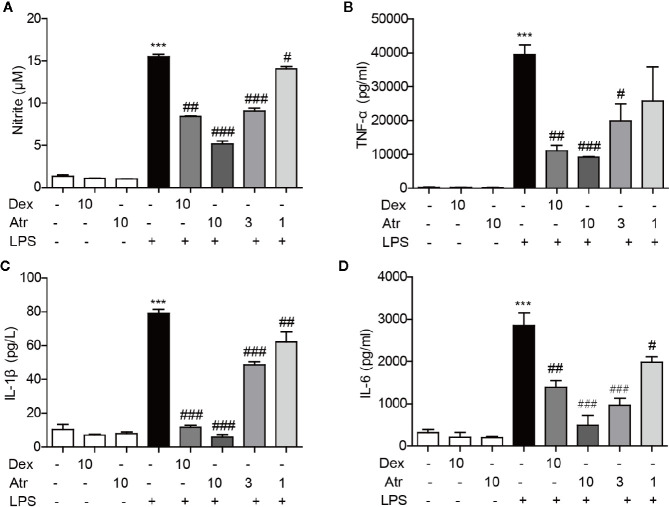
Effects of atractylodin (Atr) on cytokine production in LPS-stimulated BV-2 cells. **(A)** Nitrite was detected using a Griss reagent kit; **(B)** TNF-α, **(C)** IL-1β, **(D)** IL-6 protein levels were measured by ELISA. dexamethasone (Dex) was used as positive control. Data are expressed as mean ± SEM. ***P < 0.001 compared with the control group, ^#^P < 0.05, ^##^P < 0.01, ^###^P < 0.001 compared with the LPS group.

LPS was used to activate BV-2 cells to further assess the anti-inflammatory effects of atractylodin. Further research indicated that atractylodin (1–10 µM) inhibited nitrite generation in LPS-induced BV-2 cells in a dose-dependent manner (**Figure 6A**). Moreover, atractylodin inhibited the production of pro-inflammatory cytokines TNF-α, IL-6, and IL-1β in the cell culture supernatants in a dose-dependent manner, which were determined by an ELISA assay according to manufacturer’s instructions. Moreover, the ability to inhibit these inflammatory factors was stronger than the positive control drug dexamethasone (Dex) at a concentration of 10 µM (**Figure 6**). These results demonstrated that atractylodin significantly blocked LPS-induced pro-inflammatory mediators such as nitrite, TNF-α, IL-6, and IL-1β in microglia, which might be responsible for its anti-inflammatory activity.

### Atractylodin Increases PEA and OEA Levels in BV-2 Cells

**Figure 7:**
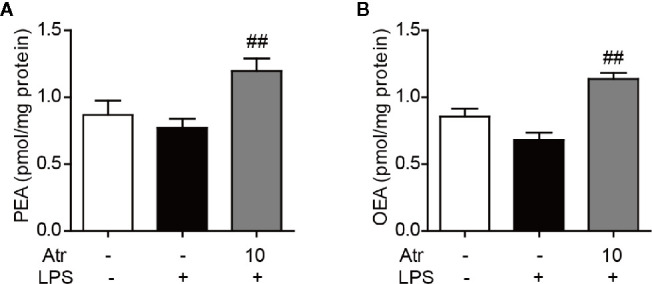
Effects of atractylodin on fatty acid ethanolamide levels in LPS-induced BV-2 cells. **(A)** PEA and **(B)** OEA levels were measured by LC-MS/MS. Data are expressed mean ± SEM. ^##^P<0.01 compared with the LPS vehicle group.

To further investigate the contribution of endogenous fatty acid ethanolamide to the anti-inflammatory effect of atractylodin, cellular PEA and OEA levels were measured by HPLC-MS/MS analysis. BV-2 cells were treated with 10 μM atractylodin following LPS activation for 6 h. PEA and OEA were extracted and analyzed according to the standard operating procedures of lipid analysis. Here, we found that cellular OEA and PEA levels slightly decreased with 1 µg/ml LPS stimulation, although there was no statistical difference. After treatment with atractylodin, both cellular OEA and PEA levels significantly increased compared to the LPS-treated control (**Figures 7A, B**).

## Discussion

It has been decades since the discovery of NAAA, and several series of chemicals have been discovered as NAAA inhibitors to date. The first generation of NAAA inhibitors was reported by Lambert’s and Ueda’s groups, which were based on the development of substrate analogs such as N-cyclohexanecarbonyl-pentadecylamine (CCP) (Tsuboi et al., 2004). A series of pyrrolidine compounds was also reported by our research group with a low micromolar potency against NAAA and reduction in cytokine production in LPS-induced macrophages (Li et al., 2012). Makriyannis’s research group developed a series of isothiocyanate-based NAAA inhibitors, and the representative compound AM9053 counteracted murine colitis. However, the highly reactive isothiocyanates scaffold might lead to off-target effects (Alhouayek et al., 2015). The second class of potent NAAA inhibitors were β-lactone and β-lactam compounds reported by Piomelli and co-workers. These chemicals showed selective inhibitory potency but with a poor plasma stability, which restricted its systematic administration (Bandiera et al., 2014; Petracca et al., 2017). Based on the structure of pyrrolidine derivatives, our research group identified a class of novel inhibitors with oxazolidone moiety, represented by F96 and F215, with nanomolar potency against NAAA (Yang et al., 2015; Li et al., 2017; Ren et al., 2017; Wu et al., 2019; Zhou et al., 2019). Migliore et al. reported third-generation NAAA inhibitors based on a benzothiazole-piperazine scaffold that is stable in mouse plasma and liver microsomes(Migliore et al., 2016). Despite great efforts in the development of NAAA inhibitors, medicinal chemistry diversity has remained low.

Phytochemicals from Chinese herbs possess broad therapeutic potential. Approximately 23.5% of approved drugs are natural products or derivatives of natural constituents (Newman and Cragg, 2020). Many natural products have good activity on cells and animal models, but most of the mechanisms of action are unclear, thereby limiting their use. Here, we first screened five NAAA inhibitors with different activities from a small library of natural products. We are particularly interested in atractylodin because of its potent inhibitory effect on NAAA and high activity in LPS-induced BV2 cells. Atractylodin is a potent, rapid, reversible, and competitive NAAA inhibitor. To our knowledge, atractylodin is the most potent natural chemical with an IC_50_ value of 2.81 ± 0.51 µM in NAAA inhibition, which is more potent than the reported NAAA inhibitors diacerein, a prodrug of the natural products rhein (IC_50_ = 7.2 ± 1.10 µM) (Petrosino et al., 2015) and fucoxanthinol from brown seaweeds (IC_50_ = 12.75 ± 1.12 µM) (Jin et al., 2020).

The crystal structure of NAAA has recently been reported by the Nagar group (Gorelik et al., 2018). NAAA belongs to the N-terminal nucleophile (Ntn) superfamily and is activated by self-proteolysis at Cys126 in human NAAA. This self-proteolysis exposes the narrow and relatively straight substrate-binding cavity, which comprises hydrogen bonds between Cys126 and Arg300, as well as Asn287 and Asp145 (Gorelik et al., 2018). The results of docking studies showed that atractylodin occupies the catalytic pocket of NAAA and engages in hydrophobic-related interactions with Trp181 and Leu152 of human NAAA, hiding the entrance of substrate PEA. Leu152 is critical to NAAA activity because when it was mutated to Phe152, NAAA activity decreased to 20% or even lower (Gorelik et al., 2018). Therefore, the discovery of atractylodin, a novel structure for NAAA inhibition, provides suitable hydrophilic head groups for the design of a novel class of NAAA inhibitors. More importantly, the polyethylene alkyne side chain in this natural product exhibits relatively rigid features, indicating more suitable adaptation for the straight and narrow binding pocket. The moiety possibly replaces the soft and long alkyl chains that are common in previously reported NAAA inhibitors, thereby improving its excessive hydrophobic feature and metabolic activity. These results may facilitate the development of more potent inhibitors targeting this enzyme.

Recent studies have demonstrated NAAA inhibition, which may be potentially utilized in controlling inflammatory responses (Pontis et al., 2016; Tuo et al., 2017; Bottemanne et al., 2018). NAAA, which is expressed in microglial cells, regulated microglial activation and thus may be potentially utilized in various CNS diseases, including neuropathic pain, Alzheimer’s disease (AD), Parkinson’s disease (PD), and Huntington’s disease (HD) (Li and Barres, 2018). The activity of these compounds to inhibit LPS-induced nitrite production in BV-2 cells is roughly related to NAAA activity. The roots of *Atractylodes lancea* have been historically used as TCM against rheumatic diseases and digestive disorders (Yu et al., 2017) in China. Several studies have attempted to elucidate the anti-inflammatory mechanisms of atractylodin; however, its molecular target remains unclear (Tang et al., 2018; Chuang et al., 2019; Jeong et al., 2019; Lyu et al., 2019). Our results show that atractylodin modulates PEA and OEA levels and suppresses cytokines in LPS-activated microglia in a dose-dependent manner through NAAA inhibition.

Although this study does not completely resolve the problems encountered in the development of NAAA inhibitors such as the lack of chemical diversity, atractylodin provides a polyethylene alkynes fragment, especially the conjugated diacetylenes moiety, which could be used in the design and synthesis of novel NAAA inhibitors. The induction of conjugated diacetylene structures could resolve the problem that most of the reported NAAA inhibitors are too flexible in structure. It could also match the flat and narrow characteristics of NAAA catalytic pocket better. At the same time, this research study has also showed that the NAAA enzyme might be one of the potential effective targets of atractylodin. By inhibiting the NAAA protein, atractylodin could exert a certain anti-inflammatory effect. These results may also guide future research investigations on the clinical anti-inflammatory and analgesic effects of the TCM Changzhu and its volatile oil. More derivatives of atractylodin should be synthesized for further structure activity relationship (SAR) research, and further mechanism studies have to be conducted to explore its detailed signaling pathway and to identify direct targets.

## Data Availability Statement

The raw data supporting the conclusions of this article will be made available by the authors, without undue reservation.

## Author Contributions

LY, ZW, and YQ designed the research. LY, CJ, and YL performed the research. HZ and FZ established the docking model. FH and JR performed FAEs extraction and quantification. LY, LL, ZW, JR, FH, and YQ analyzed the data. LY, YQ, LL, and ZW drafted the paper. All authors contributed to the article and approved the submitted version.

## Funding

This work was supported financially by the Fujian Provincial Natural Science Foundation (No. 2018J05142), the Scientific Research Foundation of Third Institute of Oceanography, Ministry of Natural Resources (No. 2016006, No.2020010), National Natural Science Foundation of China (No. 81901133).

## Conflict of Interest

The authors declare that the research was conducted in the absence of any commercial or financial relationships that could be construed as a potential conflict of interest.
